# The Role of Nutritional Aspects in Food Allergy: Prevention and Management

**DOI:** 10.3390/nu9080850

**Published:** 2017-08-09

**Authors:** Alessandra Mazzocchi, Carina Venter, Kate Maslin, Carlo Agostoni

**Affiliations:** 1 Pediatric Intermediate Care Unit, Fondazione IRCCS Ospedale Ca’ Granda-Ospedale Maggiore Policlinico, Department of Clinical Sciences and Community Health, University of Milan, 20122 Milan, Italy; carlo.agostoni@unimi.it; 2 Section of Allergy and Immunology, Children’s Hospital Colorado, University of Colorado, Aurora, CO 80045, USA; carina.venter@childrenscolorado.org; 3 MRC Lifecourse Epidemiology Unit, University of Southampton, Southampton SO16 6YD, UK; maslinkate@gmail.com

**Keywords:** food allergy, children, diet diversity, adequate nutrition

## Abstract

The prevalence of food allergy in childhood appears to be increasing in both developed and transitional countries. The aim of this paper is to review and summarise key findings in the prevention and management of food allergy, focusing on the role of dietary components and nutritional habits in the development and optimal functioning of the immune system. Essential fatty acids, zinc and vitamin D are likely to enhance the anti-inflammatory and antioxidative barrier and promote immunologic tolerance. Additionally, nutritional components such as pre- and probiotics represent a novel research approach in the attempt to induce a tolerogenic immune environment. For all these reasons, the traditional avoidance diet has been, in recent years, completely reconsidered. New findings on the protective effect of an increased diversity of food introduced in the first year of life on allergic diseases are consistent with the hypothesis that exposure to a variety of food antigens during early life might play a role in the development of immune tolerance. Accordingly, therapeutic (and even preventive) interventions should be planned on an individual basis.

## 1. Introduction

Food allergy (FA) represents a substantial health problem in childhood. The prevalence appears to be increasing in both developed and transitional countries, however a true increase has been difficult to demonstrate [1]. Over 90% of food allergies are caused by eight common allergens; namely: eggs, peanuts, cow’s milk, soy, nuts, shellfish, fish, or wheat [2]. On the whole, food allergy affects approximately 6% of infants younger than three years [2], and prevalence decreases over the first decade. The cumulative incidence of food hypersensitivity over a 10-year period is 6.7% (95% CI: 5.2–8.4%); 3.0% (95% CI: 1.8–4.2%) had IgE-mediated food allergy and 0.6% (95% CI: 0.07–1.3%) had non-IgE-mediated food allergy/food intolerance [3]. A systematic review from the European Academy of Allergy and Clinical Immunology concluded that food allergy prevalence in Europe ranges between 0.1 and 6.0% [4]. The Institute of Medicine report states that the prevalence of food allergies in children ranges between 1.1 and 10.4% [1]. Food-allergic infants commonly present with symptoms and signs of atopic eczema, gastrointestinal symptoms and/or recurrent wheezing [5]. Diet plays a crucial role in both the prevention and management of food allergy. A number of factors, including the maternal diet, the microbiome and early life feeding, have been investigated for the prevention of allergic diseases [6]. The aim of this paper is to review and summarise key findings in the prevention and management of food allergy, with particular reference to nutrients of concern (fats, micronutrients), gut flora (including the role of pre- and probiotics), early-life feeding and formula choice in cow’s milk allergy.

## 2. Prevention of Food Allergy: The Role of Nutrition in the Development and Optimal Functioning of the Immune System

Allergy results when there is a breakdown in normal “tolerance” mechanisms, which leads to inappropriate and detrimental immune responses to normally harmless substances, including food allergens such as cow’s milk protein, eggs, nuts, or shellfish [7]. At birth, the immune system is immature, but it develops with age, antigen stimulation, and appropriate nutrition [8]. In addition, bacterial colonisation occurs during the first weeks of life, and interactions between intestinal flora and the developing mucosa result in further development of immune responses and oral tolerance [7].

Nutrition plays a key role in the development, maintenance, and optimal functioning of immune cells. Nutrients, such as zinc and vitamin D and nutritional factors, such as pre- and probiotics, can influence the nature of an immune response and are important in ensuring appropriate functioning of the immune system, as described in the paragraphs below.

### 2.1. Fat

Appropriate fat intake may become seriously compromised in allergen-restricted diets and may be further influenced by the “westernized” dietary practices. The role of fat on the immune system can be divided into the role of saturated vs. unsaturated fats and the particular role of the essential fatty acids.

#### 2.1.1. Saturated vs. Unsaturated Fats

It has been reported that typical western diets rich in protein and saturated fat and low in complex carbohydrates may negatively affect the diversity of the gut microbiome [9]. This was supported by David et al. [10], showing that an animal-based diet high in protein and fat, with very little fibre intake, resulted in increased abundance of bile-tolerant microorganisms (*Alistipes*, *Bilophila*, and *Bacteroides*) and decreased levels of Firmicutes that metabolize dietary plant polysaccharides (*Roseburia*, *Eubacterium rectale*, and *Ruminococcus bromii*) within a five-day period. A recent review also concluded that the amount, type (e.g., unsaturated vs saturated), and mixture of dietary fats can dramatically shift gut microbial community membership and function [11]. In addition, high fat, high sugar diets also affect the gut barrier function in mice, as demonstrated by high horseradish peroxidase (HRP) influx, lower portal vein endotoxin levels and decreased goblet cell numbers [12]. The gut barrier function may be permanently affected in non-IgE mediated food allergies, and temporarily affected during allergen exposure in IgE mediated food allergies [13,14].

#### 2.1.2. Essential Fatty Acids (EFAs)

EFAs are important immune regulators. Linoleic acid (LA), the parental *n-*6 polyunsaturated fatty acid (PUFA), is converted into arachidonic acid (AA) by fatty acid elongase and desaturase, and subsequently may give origin to pro-inflammatory and pro-allergic lipid mediators, whose collective name is eicosanoids [15]. In contrast, α-linolenic acid (ALA), an *n-*3 PUFA, is converted in the mammalian body to eicosapentaenoic acid (EPA) and docosahexaenoic acid (DHA), which are subsequently converted into anti-inflammatory and/or pro-resolving lipid mediators (such as resolvins and protectins). EPA forms the precursors of the 3 series of prostaglandins and the 5 series of leukotrienes, which are biologically less powerful than the corresponding derivatives which form the *n-*6 compounds. Because *n-*3 and *n-*6 PUFAs compete for the same metabolic pathways, an increase of *n-*3 PUFA, parallel to a decrease of *n-*6 PUFA intake, might theoretically reduce the onset of human immunologic conditions, including allergies, thanks to the replacement of AA with EPA and DHA in the membranes of inflammatory cells. EFA, including long-chain PUFAs, may be consumed as part of the normal diet through breast milk, formula and food, or as supplements at any stage in the life cycle [15].

The fatty acid status is of particular concern in infants and children. Essential fatty acids (EFA) promote the renewal of the protective hydrolipidic film layer of the skin and, accordingly, an altered EFA metabolism has been associated with the pathogenesis of atopic dermatitis (AD). Moreover, the clinical spectrum of EFA deficiency may range from mild skin irritation to life-threatening conditions [16].

In spite of intensive research in the field, a recent systematic review [17] concerning the role of dietary PUFAs in the development of allergy shows that PUFA supplementation in infancy seems not to affect infant incidence, childhood incidence or childhood prevalence of food allergy (GRADE level of evidence: very low; GRADE, Grading of Recommendations Assessment, Development and Evaluation) in infants up to two years of age, even taking into account a moderate heterogeneity between studies that reported infant incidence of food allergy (3 studies; 915 infants; RR (risk ratio) 0.81, 95% CI 0.56–1.19%, *I*^2^ (fraction of variance due to heterogeneity) = 63%; RD (risk difference) 0.02, 95% CI: 0.06–0.02%, *I*^2^ = 74%). However, while well-documented immunomodulatory effects of *n-*3 PUFAs (both in vitro and in vivo) highlight the potential role in preventing and treating allergic disease, larger longitudinal intervention studies are clearly warranted to confirm this observation [18].

### 2.2. Zinc

Children with food hypersensitivity have increased amounts of mastocytes, eosinophils and neutrophils in the digestive tract. Persistent exposure to allergen can lead to chronic inflammatory changes of mucous membrane and increased production of reactive oxygen species (ROS) [19]. Excess ROS should be neutralized by components of the antioxidative barrier. Therefore, all disturbances of enzymatic and non-enzymatic mechanisms of this barrier lead to many unfavourable reactions, including oxidation of cell membrane lipids. Zinc is an essential trace element and it is needed for various cellular functions; specifically, it is a cofactor of many enzymes, including superoxide dismutase (SOD), which play an important role in maintaining the oxidative-antioxidative balance. A study performed in 134 children with food allergy, aged 1 to 36 months, showed that children with food allergy had significantly lower concentrations of zinc, and therefore a weakened antioxidative barrier [19]. To our knowledge there are no randomized controlled trials (RCTs) investigating zinc supplementation and allergic outcomes.

### 2.3. Vitamin D

The classical role of Vitamin D is, in fact, related to calcium homeostasis and bone health. However, over the last decade, the effects of vitamin D on the innate and adaptive immune system have been investigated and expanded [20]. The active form of the vitamin, i.e., 1,25(OH)2D (calcitriol), has effects on epithelial cells, T cells, B cells, macrophages and dendritic cells. It stimulates innate immune responses by enhancing the chemotactic and phagocytotic responses of macrophages, as well as the production of antimicrobial proteins such as cathelicidin. This action plays a role in maintaining mucosal integrity by stimulating junction genes. Nevertheless, the potential effect of vitamin D on Th1/Th2 adaptive immune response is of interest and related to food allergy [21,22,23]. Almost all cells of the adaptive immune system express the vitamin D receptor, making them also capable of being vitamin responsive. When specifically considering a potential role for vitamins in food allergy, vitamin D has been shown to affect several mechanisms that promote immunologic tolerance, including T regulatory cell function and the induction of tolerogenic dendritic cells. However, clinical trials on vitamin D supplementation in children and the possible role in preventing food allergy are lacking. A systematic review of vitamin D supplementation for the prevention of allergic diseases found no evidences about the protective role of this nutrient in children, but the currently available data are poor [24].

### 2.4. The Role of Prebiotics, Probiotics and Microbiota in the Prevention of Food Allergy

The innate immune system has the ability to modulate adaptive immune responses to food proteins. Therefore, the type of gastrointestinal microbiota of the newborn and the preservation of intestinal permeability is crucial for preventing the development of food allergies. The dietary modulation of nutritional factors through pre-, pro- and synbiotic preparations represent a novel research hypothesis and a challenge for dietitians and paediatric allergists. The modulation of the immune system using functional foods is a promising research hypothesis in the attempt to induce a tolerogenic immune environment [16].

#### 2.4.1. Prebiotics

Prebiotics have been defined as “non-digestible food components that beneficially affect the host by selectively stimulating the growth and/or activity of one or a limited number of bacteria in the colon and thereby improving host health”, and recently redefined as “a selectively fermented ingredient that allows specific changes, both in the composition and/or activity in the gastrointestinal microbiota that confers benefits” [25]. In December 2016, the panel of experts convened by the International Scientific Association for Probiotics and Prebiotics (ISAPP) suggested a new definition, i.e., “a substrate that is selectively utilized by host microorganisms conferring a health benefit” [26]. Based on the body of available evidence, the Guidelines for Atopic Disease Prevention (GLAD-p) panel concluded that it is likely that prebiotic supplementation in infants reduces the risk of developing recurrent wheezing and possibly also the development of food allergy. However, there is very low certainty that there is an effect of prebiotics on other outcomes, other than an indirect effect due to its effect on the microbiome. In fact, their activity can be affected by many individual factors, (e.g., host’s microbiota or the genetic predisposition to diseases). Environmental factors such as diet or antibiotics can also influence the use of prebiotics [26].

#### 2.4.2. Probiotics

Probiotics are living microorganisms that have been proposed as immune-modulators of the allergic response by affecting phagocytosis and production of pro-inflammatory cytokines, and thus have been advocated as therapeutic and preventive interventions for allergic diseases [27]. They are present in everyday food (not only in yoghurt or fermented milk, but also in cheese—either hard or soft—and also in less expected sources such as kefir, miso soup or tempeh) and they are a common exposure in almost everyone’s life [27]. The probiotic effects of complex oligosaccharides in human milk promote the establishment of a bifidogenic microbiota which, in turn, induces a milieu of tolerogenic immune responses to foods. Earlier studies suggested a positive effect of probiotic interventions on atopic dermatitis, but meta-analyses have failed to confirm it.

The new World Allergy Organization (WAO) guidelines determined that it is likely that probiotic supplementation in infants reduces the risk of developing eczema and suggested that probiotics should be recommended in mothers of high-risk infants and in infants at high risk of allergic disease, where “high risk for allergy in a child” is defined as having a biological parent or sibling with an existing or history of allergic rhinitis, asthma, eczema, or food allergy [27]. The recommendations are conditional, and based on very low-quality evidence, with no specific recommendation regarding strains, dose, treatment duration etc.

In terms of tolerance development in those with established food allergy, one study from Australia performed oral immunotherapy (OIT) to peanut in combination with Lactabillus GG, showing that 89.7% of the study participants in this arm were desensitized to peanut. The authors speculate that this protective effect may be seen because of the possible effect of the probiotic on T regulatory cells [28]. Further scientific confirmation is required to include probiotics and prebiotics in the therapeutic plans. Practical implications and how this should be incorporated in advising food allergy sufferers are also unclear in terms of advising regular intake of foods high in short-chain fructo-oligo saccharides, fermented foods and yoghurts.

## 3. The Role of Allergen Intake and Dietary Diversity in Prevention of Food Allergy

### 3.1. Allergen Intake

Measures to prevent allergy and food allergy have traditionally included maternal allergen avoidance during pregnancy and/or lactation, periods of exclusive breast feeding and avoidance of potential allergens, including food and environmental antigens, during the first year of life and beyond [29]. The value and significance of food avoidance for preventive purposes has been completely reconsidered in recent years.

On the contrary, an ideal age to introduce potentially allergenic foods into an infant’s diet has been debated for the past 2 decades, particularly in high-income countries where allergic disease has become highly prevalent. Initial approaches to primary prevention of food allergy largely focused on “avoidance” strategies. In 2000 [30], practice guidelines generally recommended that allergenic foods (such as egg, cow’s milk, and peanut) be avoided during the first 1 to 3 years of life. As data accumulated from both observational studies and experimental models, it became apparent that avoidance practices may not be beneficial.

Given the increasing interest in the role of time of introduction of allergic food into the infant diet (the so-called “window of opportunity”) and the risk of allergic diseases, intervention trials evaluating the intake of food, as milk, egg, peanuts, etc., during the first year of life have been performed.

For instance, a recent RCT found no evidence that regular egg intake from age 4 to 6.5 months substantially alters the risk of egg allergy by age 1 year in infants who are at hereditary risk of allergic disease and had no eczema symptoms at study entry [31]. These findings are generally supportive of other data in high-risk patients showing a risk-reducing benefit for early egg introduction, and risk-reducing benefit for early peanut introduction [32]. The EAT study [33] also showed a reduced risk in the general population using the per protocol analysis, but not the intention to treat analysis. For peanut, clinical practice guidelines in the US have incorporated these findings and do recommend early peanut introduction in the first year of life for high- and standard-risk children [34]. However, despite some evidence for early introduction of egg, the US guidelines only made recommendations regarding peanut intake, and concluded that there was not enough evidence to suggest early introduction of egg. Surprisingly, the UK COT report [35], published very recently, suggested that all foods should be introduced after a period of exclusive breast feeding from 6 months and that there is no need to introduce peanut or egg differently from other foods. It seems as if despite the data from recent RCTs on peanut and egg, the weaning debate will continue, as there is still no consensus about the age of introduction of these foods. The only consistent messages are: start weaning once the infant is developmentally ready; don’t delay introduction of allergens: once they are introduced into the diet, continue to feed them.

### 3.2. Diet Diversity and Other Related Factors

#### 3.2.1. Dietary Diversity

Recent findings on the protective effect of an increased diversity of food introduced in the first year of life on allergic diseases (asthma, atopic dermatitis, food allergy and atopic sensitisation) are consistent with the hypothesis that exposure to a variety of food antigens during early life might be important for the development of immune tolerance [36,37,38].

The microbiome plays an important role in ensuring the gut wall integrity and regulation of the immune system. Diet diversity has been shown to reduce allergic diseases [36,38]. It may well be that the more diverse diet leads to a more diverse microbiome [39], and that natural microbial load of food enhances this process [40]. This in turn may improve the gut wall integrity and regulation of the immune system, but human trials are needed to confirm this theory.

#### 3.2.2. Food Production

Food production and cooking methods, inclusive of canning, putting food in pouches, producing “ready to eat” foods, may affect the natural microbioal load of food and hence the immune system as it may affect the microbiome and, possibly, the allergic response.

Lang et al. [40] reported that the microbial load of different diets (e.g., USA diet vs. vegan diet) differs due to the foods excluded and cooking methods used. Chaturvedi, et al. [41] reported that the natural microbial load of fruits and vegetables differ between groups from a different socio-economic status. In addition, Venter and Maslin reported an association between an increase in baby food sales and allergic diseases [42], underlining that commercial baby foods are sterile and that the diversity of ingredients and nutrient content is variable. All these factors highlight that the foods we eat (irrespective of their nutrient content) may affect the immune system and perhaps development and management of allergic diseases.

#### 3.2.3. Healthy Diet

It is unclear at present what a “healthy diet” in terms of allergy prevention and management means, and if a healthy diet as we know it (20% protein, 50% carbohydrate, 30% fat) has any relevance in allergy prevention. Currently, either the healthy eating index [43] or a Mediterranean-style diet [44] is being used as a proxy measure for healthy eating. Research using the healthy eating index tool, specific to the pregnancy diet, found no association between overall healthy eating score and recurrent wheeze in infants at the age of 3 years [43], and this was confirmed in another study by Moonesinghe et al. focusing on eating patterns in pregnancy and allergic diseases [45]. In addition to these two studies, two review papers addressed the issue of the Mediterranean diet on allergy prevention. Venter et al. summarised studies during pregnancy [46]. Three observational studies have investigated the role of the Mediterranean diet on allergy outcomes. One study showed a possible increased risk for the infant to develop allergic disease [47], one showed a reduction in wheeze [48], and another study showed no effect on allergy prevention [49]. Mediterranean style eating patterns shows more promising effects with reduction in asthma/wheezing symptoms seen but no effect on other allergic symptoms [44]. More studies are therefore needed with well-defined criteria for healthy eating to study its effect on allergy prevention.

#### 3.2.4. Other Factors

More recently, the role of advanced glycosylated end products in food and the direct effect on the Th2 immune system and the microbiome has been described [50]. One mouse model study also questioned the role of emulsifiers on the gut microbiome. This study showed that a diet high in emulsifiers destroyed the epithelial mucous layer in the gut, altered gut microbial composition and promoted inflammation [51].

## 4. The Role of Diet in the Management of Food Allergy

The cornerstone of the nutritional management of food allergies is an individualized allergen avoidance management plan. In children, the main goals are to prevent the occurrence of acute and chronic symptoms by avoiding the offending food(s), whilst providing an adequate, healthy and nutritionally balanced diet and maintaining optimal growth; ideally, under the guidance of a trained dietitian [52]. Complete avoidance of the allergen is still required by some, but latest developments in food allergy have indicated that some individuals with food allergies tolerate baked forms of milk and egg [53]. Additionally, complete avoidance of all nuts is not necessarily recommended anymore, and only those nuts reacted to should be eliminated from the diet [54]. In addition to nutritional consequences of food allergy, it is known that children and families with food allergies experience a decreased quality of life across a number of domains, which can create anxiety and lead to avoidance of social situations [55,56,57,58]. Hence, it is suggested that liberalisation of the diet, when appropriate and safe, will increase both quality of life and nutritional intake.

### 4.1. Cow’s Milk Allergy

Exclusion of any food group can result in a nutritionally deficient diet, but the elimination of milk and products in infancy is particularly likely to cause nutritional deficiencies [59] and deserves special emphasis. Cow’s milk proteins (CM) are among the first foods introduced into an infant’s diet, and accordingly they represent one of the first and most common causes of food allergy in early childhood. Cow’s milk allergy generally requires a strict exclusion diet, usually for the first year of life. This exclusion of a main food group occurs at a critical time in the development of food preferences and eating habits. The management of CMA (cow’s milk allergy) in infants and young children requires individualized advice regarding avoidance of cow’s milk, including advice to breastfeeding mothers and/or guidance on the most appropriate specialized formula or milk substitute [60]. In many cases, micronutrient supplements will also be required; however, their usage is not always intuitive with both under- and over-supplementation occurring [61].

Cow’s milk proteins could induce an allergic reaction: in particular beta-lactoglobulin (BLG), included in the whey fraction, is not present in human milk, and is therefore is considered the principal component involved in the etiology of the disease. During the production of infant formula, only the processes of extensive hydrolysis, ultrafiltration or an enzymatic cleavage result in truly hypoallergenic formulas [16].

#### 4.1.1 Choice of Formula in CMA

The nutritional value of a milk substitute must be taken into account at ages lower than 2 years of life, when such a type of food is needed and may represent the only source of nutrients in the first months of life [16]. As breast milk composition differs both in component ratios and structure from other milks, the composition of infant formula should serve to meet the particular nutritional requirements and to promote normal growth and development of the infants for whom they are intended [62,63]. When a replacement formula is needed, allergologists can avail themselves of different types of formula [64]. The alternative formulas considered for CMA are extensively hydrolysed whey or casein formula (eHWF or eHCF), and amino acid-based formula (AAF), which are considered to be of low antigenic potential and are therefore preferred in highly allergic children. The unpalatable taste of hydrolysed formulas has often been associated with reduced intakes and a consequent growth faltering in infants fed these types of formula, particularly in the first year of life [59].

In recent years, an alternative explanation has been proposed based on the content of free amino acids (FAAs) in hydrolysed formulas, added to complete their biologic value. Glutamic acid, in particular, has been suggested to downregulate appetite during feeding by interacting with specific receptors in the oral cavity and gastrointestinal tract. However recent studies have shown no negative effect of feeding AA formulas in infants; on the contrary, they may be beneficial for growth [65].

Other studies have demonstrated that dietary management with extensively hydrolysed casein-based formula (eHCF) supplemented with the probiotic Lactobacillus rhamnosus GG (LGG) results in a higher rate of tolerance acquisition in infants with CMA than in those treated with eHCF without supplementation or with other non-casein-based formulas. The mechanistic basis for this effect could be the possible influence of eHCF+LGG on the strain-level bacterial community structure of the infant gut [66]. However, randomised controlled trials to date have not yielded sufficient evidence to recommend probiotics for the primary prevention of allergic disorders. Indeed, the Nutrition Committee of the European Society for Paediatric Gastroenterology Hepatology and Nutrition (ESPGHAN) does not support routine supplementing with probiotics in infant formulas [67].

Soy protein-based formula may be an option in infants older than 6 months who do not accept the bitter taste of an eHCF, or in cases in which the higher cost of an eHCF is a limiting factor [68]. However, soy formulae have nutritional disadvantages. Absorption of minerals and trace elements may be lower because of their phytate content. They also contain appreciable amounts of isoflavones, with a potentially weak estrogenic action that can lead to high serum concentrations in infants. Also, the possible derivation from genetically modified soy should be considered. Hence, the European Society of Paediatric Gastroenterology, Hepatology, and Nutrition (ESPGHAN) and the American Academy of Pediatrics (AAP) recommend that cow’s-milk-based formulas should be preferred over soy formulas in healthy infants, and soy protein-based formulas should not usually be used during the first 6 months of life [68].

Other mammal’s milks, those of, goats, ewes, mares, donkeys, or camels, have been proposed as substitutes in the management of CMA in infants and children, but are NOT recommended, due to either nutritional issues, cross-reactions or both. The Diagnosis and Rationale for Action against Cow’s Milk Allergy (DRACMA) guidelines state that milk allergens of various mammalian species cross-react [16]. The greatest homology is found between cow’s, buffalo’s, sheep’s and goat’s milk proteins. Proteins in their milks have less structural similarity with pig, horse, donkey, camel and dromedary. Goat’s, buffalo’s and ewe’s milk are particularly not recommended by the World Allergy Organization due to cross-reactivity with cow’s milk [16]. The tolerance of other mammalian milks needs to be further investigated in clinical trials, and there are some concerns about their chemical composition and sanitation. In conclusion, either amino acid-based formulas or eHCFrepresent the most available solutions for allergic infants who are no longer breast-fed. The therapeutic interventions should therefore be indicated on an individual basis.

## 5. Conclusions

Food allergy represents a significant health burden at either an individual and population level worldwide. Recent guidelines for the prevention of food allergies advocate that there is no need to delay the introduction of allergenic foods once weaning has commenced. In terms of food allergy management (end even prevention), individualised strategies should be implemented. These strategies will include developmental readiness to be weaned, prevalence of particular food allergies in certain countries, family eating patterns and availability of physician and dietetic care.

Care should be taken to ensure adequate intake of nutrients, particularly in relation to cow’s milk allergy, when selecting a suitable hypoallergenic formula. There is emerging evidence regarding the role of fats (particularly EFAs), pre-/probiotics, commercial foods, healthy eating and micronutrients on food allergy. A better understanding of how nutrients and other aspects of food, food patterns and food preparation may affect the immune system and allergy outcomes is required to best advise those at risk of developing food allergies and those with current food allergies.
